# Sixteen isotropic 3D fluorescence live-imaging datasets of gastrulation in the red flour beetle *Tribolium castaneum* (Coleoptera: Tenebrionidae)

**DOI:** 10.1038/s41597-026-08047-9

**Published:** 2026-09-11

**Authors:** Franziska Krämer, Stefan Münster, Frederic Strobl

**Affiliations:** 1 Physical Biology / Physikalische Biologie (IZN, FB 15), Buchmann Institute for Molecular Life Sciences (BMLS), Cluster of Excellence Frankfurt – Macromolecular Complexes (CEF – MC), Goethe-Universität – Frankfurt am Main (Campus Riedberg), Max-von-Laue-Straße 15, D-60438 Frankfurt am Main, Germany; 2 Philipps-Universität Marburg, Fachbereich Biologie, Molekulare Tierphysiologie, Marburg, Germany

## Abstract

Gastrulation is a pivotal phase of early embryogenesis during which initially uniform cells undergo coordinated movements and fate specification to establish the basic body plan. Although previous studies have advanced our understanding of gastrulation in the red flour beetle *Tribolium castaneum*, quantitative morphogenetic data remain limited. Here, we present the second Systematic Live Imaging Collection of Embryogenesis (SLICE-2), consisting of sixteen isotropic 3D fluorescence live imaging datasets that document gastrulation and early germband elongation in *Tribolium*. Imaging was performed using light sheet fluorescence microscopy in conjunction with a homozygous transgenic line that expresses mEmerald-labeled nanobodies against histone H2A/H2B. Embryos were recorded along four directions, and the resulting axial image stacks were subjected to multiview fusion to obtain isotropic 3D images of entire embryos with reduced shadowing artifacts. The collection includes weighted-average fusion and fusion-deconvolution derivatives, and nuclei segmentation data are available for selected developmental stages. SLICE-2 expands the microscopy data resources for *Tribolium*, enabling comprehensive analyses of gastrulation dynamics while also providing benchmark data for image processing, segmentation, and modeling approaches.

## Background & Summary

Understanding how a single cell gives rise to a complex multicellular organism is a central goal of developmental biology^1^. Gastrulation, a defining event of early embryogenesis, involves extensive tissue reorganization during which initially uniform cells undergo coordinated movements and begin to adopt distinct identities. In triploblastic organisms, this process establishes the three germ layers – ectoderm, mesoderm, and endoderm – from which all embryonic tissues arise^2^. The cellular mechanisms and morphogenetic dynamics underlying gastrulation vary considerably across the metazoan lineage^3,4^.

**Fig. 1:**
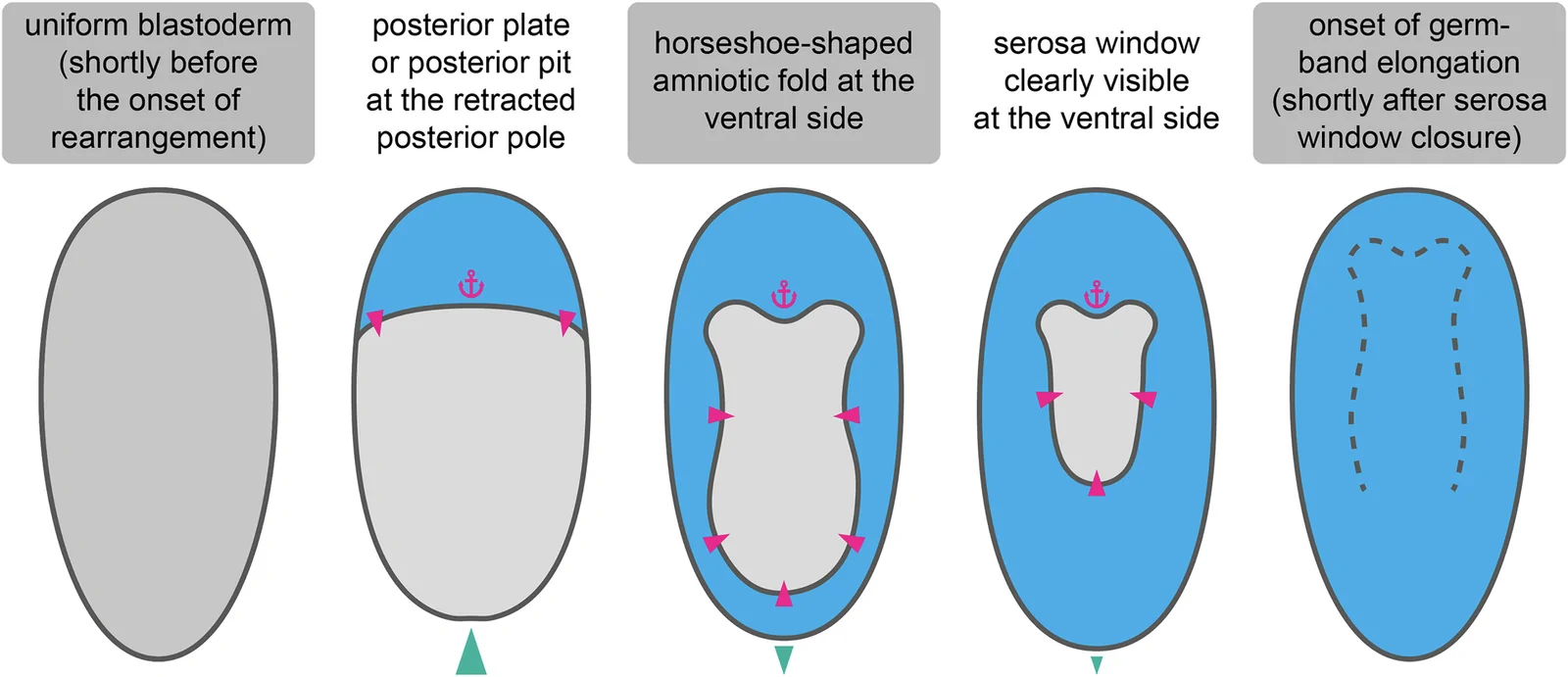
Overview of the morphogenetic sequence underlying gastrulation in *Tribolium*. Five characteristic stages are shown in ventral view. In brief, the uniform blastoderm (first panel) retracts from the posterior pole during the formation of the posterior plate/posterior pit (second panel, teal arrowhead). Blastodermal cells within a dorsally tilted anterior cap (second panel, blue region) differentiate into the serosa, which begins to spread over the yolk and the condensing embryonic rudiment (second panel, pink arrowheads). A cluster of anteroventral cells remains attached to the vitelline membrane, a process thought to contribute to the characteristic unidirectional tissue flow during gastrulation (second panel, anchor symbol). Subsequently, the serosa extends over the posterior pole and progressively expands (third panel, pink arrowheads), resulting in the formation of a horseshoe-shaped amniotic fold between the serosa and the condensing germ rudiment (third panel). Concurrently, the initial retraction of the blastoderm at the posterior pole partially reverses (third panel, teal arrowhead). As serosal expansion continues (fourth panel, pink arrowheads), the ventral serosa window emerges, while posterior retraction continues to reverse (fourth panel, teal arrowhead). Eventually, the serosa window closes, and the serosa fully envelops underlying structures, including the germband (fifth panel, dashed gray line) as well as the yolk.

The red flour beetle *Tribolium castaneum* is an important model organism in evolutionary developmental biology^5^. It combines practical advantages such as a short generation time and genetic tractability with a developmental mode considered more representative of ancestral insect developmental programs than the highly derived embryogenesis of *Drosophila melanogaster*^6^. For example, *Tribolium* exhibits short/intermediate-germ development with sequential posterior segment addition, prominent extra-embryonic membranes, externally developing thoracic appendages, and a non-involuted head morphology^7^. Previous studies have provided important insights into diverse aspects of gastrulation in *Tribolium*, including mesoderm invagination^8–10^, segment identity specification^11–16^, and the formation of extra-embryonic membranes^14,17–20^ together with its underlying biomechanical mechanisms^21,22^. Unlike *Drosophila*, which gives rise to only a single reduced extra-embryonic membrane, the amnioserosa^23^, *Tribolium* develops two distinct extra-embryonic layers, the amnion and the serosa. During gastrulation, the serosa spreads over the yolk and the condensing embryonic rudiment before closing at an anterior-ventral position, thereby enveloping all underlying structures, including the germband (Fig. 1)^14,22,24^. Despite the biological significance of these morphogenetic processes, live imaging datasets of gastrulation in *Tribolium* suitable for quantitative analyses remain scarce. For example, the fraction of blastodermal cells contributing to the serosa has only been roughly estimated at about one third, while absolute numbers remain undetermined^8,21,25^.

To expand the microscopy data resources available for *Tribolium*, we previously established the first Systematic Live Imaging Collection of Embryogenesis (SLICE-1), which documents nearly the entire embryogenesis through fluorescent labeling of diverse intracellular structures^26^. Although the SLICE-1 datasets also cover gastrulation, their suitability for comprehensive quantitative analyses remains limited due to spatially uneven image quality within individual embryos and a comparatively small number of biological replicates. Here, we present the second Systematic Live Imaging Collection of Embryogenesis (SLICE-2), comprising sixteen isotropic 3D fluorescence live imaging datasets documenting gastrulation in *Tribolium*. Data were acquired using light sheet fluorescence microscopy in combination with the AGOC{ATub’H2A/H2B°NB-mEmerald} #1 transgenic subline^26^, which expresses mEmerald-labeled nanobodies against histone H2A/H2B under the control of the constitutively and ubiquitously active *tubulin alpha 1-like protein* promoter^27^. Imaging began at the end of blastoderm formation and continued for approximately 24 hours, covering gastrulation and early germband elongation. All embryos hatched morphologically intact, all but one larva reached adulthood, and all but two adults produced progeny.

**Fig. 2:**
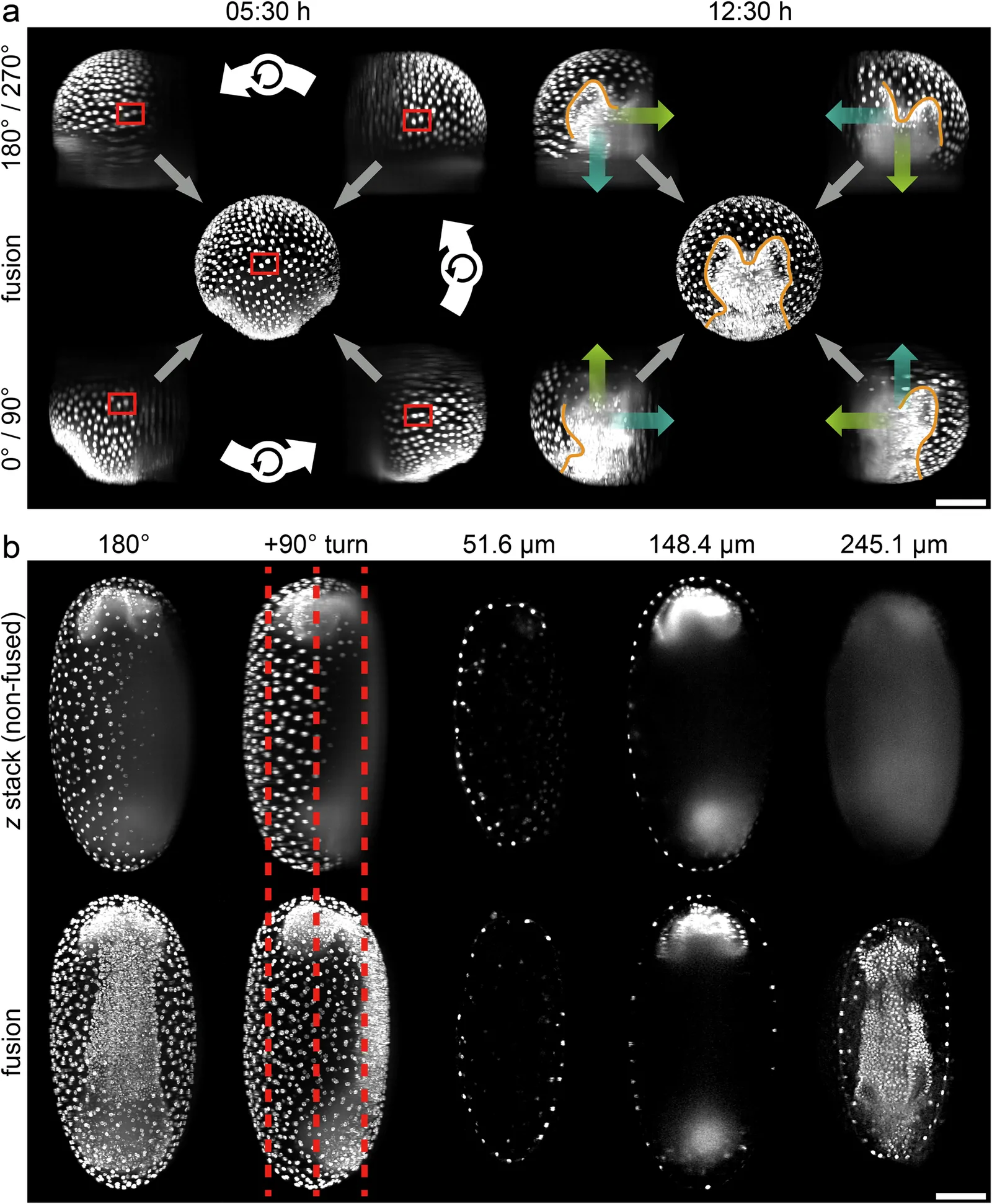
Multi-view fusion of imaging data obtained from a *Tribolium* embryo expressing mEmerald-labeled nanobodies against histone H2A/H2B. (**a**) Shown are *y* projections of *z* stacks acquired along four directions spaced 90° apart (outer panels) together with the corresponding fused 3D images (inner panels, indicated by gray arrows) at two developmental time points. At 05:30 h, white arrows indicate the acquisition order of the four directions, which were computationally rotated back by 90° to display the embryo from the same perspective in all panels. Red rectangles highlight the same two nuclei across panels. At 12:30 h, cyan and green arrows denote the illumination and detection axes, respectively. Orange outlines indicate the regions of the developing germband that are clearly visible in the respective projections, i.e. not obscured by shadowing artifacts. The full contour of the germband becomes discernible only in the fused image. (**b**) Shown are *z* projections (first column), *x* projections (second column), and optical sections (third to fifth columns) of a *z* stack with interplane interpolation together with the corresponding fused 3D image obtained at the end of the D branch of the image processing workflow. Red dashed lines mark the positions of the optical sections. The reduction of shadowing artifacts and the corresponding improvement in signal quality are most apparent at a depth of ~245 µm. Image data are from dataset DS0003. Scale bars, 100 µm.

Embryos were recorded along four directions spaced 90° apart, and the resulting image stacks were subjected to multiview fusion to obtain isotropic 3D reconstructions of the data with strongly reduced shadowing artifacts^28^ (Fig. 2). The fused images were subsequently rotated around all three spatial axes to align embryonic and image axes, cropped to standardized sizes, and adjusted in brightness and contrast. The resulting isotropic 3D images are available as two different derivatives: weighted-average fusions and fusion-deconvolutions. As an example of the analyses enabled by these datasets, superficial blastodermal nuclei at the uniform blastoderm stage and serosa nuclei after serosa window closure were segmented. This yielded 3553 ± 104 and 845 ± 129 nuclei (mean ± standard deviation), respectively, suggesting that the serosa derives from closer to one quarter of blastodermal cells rather than the previously assumed one third.

SLICE-2 complements SLICE-1 and other existing microscopy data resources for *Tribolium* by providing fused 3D images with comparatively uniform image quality across the entire embryo. This enables statistically robust and spatially comprehensive analyses of the morphogenetic processes underlying gastrulation. In addition, the non-fused *z* stacks and fused 3D images provide reference material for evaluating alternative fusion and/or deconvolution workflows^29^, while the accompanying segmentation data (exemplified in Supplementary Movies 1 and 2) can be used directly for downstream analyses and computational modeling or serve as ground truth for the development and benchmarking of semi-automated or automated segmentation frameworks^30^.

## Methods

### *Tribolium castaneum* transgenic lines and rearing

Imaging cultures of the transgenic AGOC{ATub’H2A/H2B°NB-mEmerald} #1 *Tribolium castaneum* subline^26^, which expresses mEmerald-labeled nanobodies against histone H2A/H2B under the control of the constitutively and ubiquitously active *tubulin alpha 1-like protein* promoter^27^, were maintained at population sizes of about 200–500 individuals. Cultures were reared on growth medium consisting of full grain wheat flour (SP061036, Demeter) supplemented with 5% (wt/wt) inactive dry yeast (62–106, Flystuff) in one-liter glass bottles under a 12:00 h light / 12:00 h darkness cycle at 32 °C and 70% relative humidity (DR-36VL, Percival Scientific). All animal-related experiments were approved by the institutional ethics committee of the Goethe University (Tierschutzkommission der Goethe-Universität, file number BB-20250515-St) and conducted in accordance with the German Animal Welfare Act (Tierschutzgesetz/Tierschutz-Versuchstierverordnung, based on ETS No. 123^31^ and EU Directive 2010/63/EU^32^) as well as the ARRIVE 2.0 guidelines^33^.

### Light sheet fluorescence microscopy

Light sheet fluorescence microscopy was performed using a sample chamber-based digital scanned laser light sheet fluorescence microscope (DSLM) featuring single-sided illumination and detection^34^. A dynamic light sheet was generated by rapidly scanning a collimated Gaussian laser beam using a two-axis piezo-driven scanning mirror (S-334.2SD1, Physik Instrumente GmbH & Co KG). Illumination was provided by a 488 nm / 20 mW diode laser (PhoxX 488-20, Omicron Laserprodukte GmbH) equipped with a 488 nm cleanup filter (xX.F488, Omicron Laserprodukte GmbH). Excitation light was delivered through a 2.5× NA 0.06 EC Epiplan-Neofluar objective (422320-9900-000, Carl Zeiss AG), while emission light was collected through a 10× NA 0.3 W N-Achroplan objective (420947-9900-000, Carl Zeiss AG). Detection was achieved using a 525/50 single-band bandpass filter (FF03-525/50-25, Semrock/AHF Analysentechnik AG) and a 16-bit (effectively 14-bit) high-resolution charge-coupled device camera (Clara, Andor) with a sensor pixel pitch of 6.45 µm, corresponding to an effective lateral pixel spacing of 0.645 µm. Conventionally^35^, the illumination axis was defined as *x*, the rotation axis as *y*, and the detection axis as *z*, with *y* aligned with the direction of gravity. Three micro-translation stages (M-111.2DG, Physik Instrumente GmbH & Co KG) were used for sample translation along *x*, *y*, and *z*, and a precision rotation stage (M-116.DG, Physik Instrumente GmbH & Co KG) for rotation around *y*. All *z* stacks had an axial voxel pitch of 2.58 µm, corresponding to a lateral-to-axial pitch ratio of 1:4. The effective laser power was routinely monitored using an optical power and wavelength meter (OMM-6810B and OMH-6703B, Newport).

### Embryo collection, preparation, mounting, live imaging, and retrieval

**Fig. 3:**
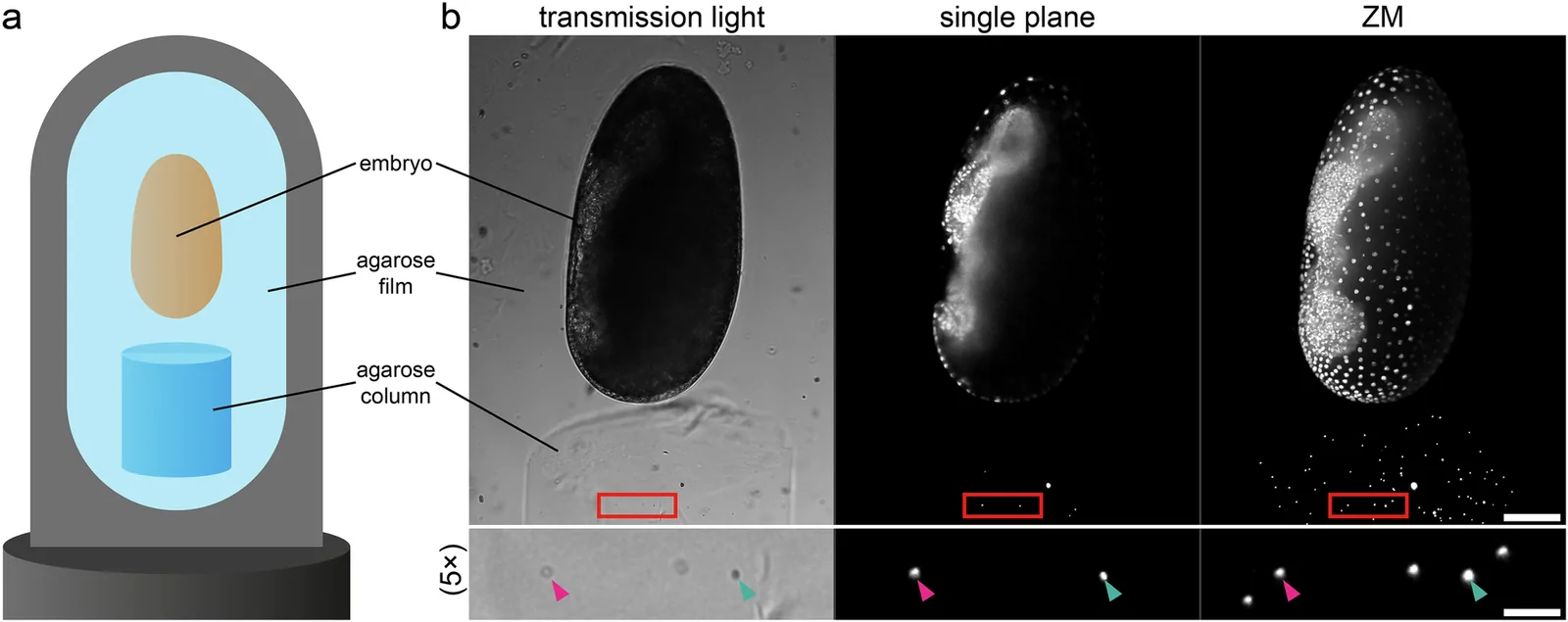
Mounting of *Tribolium* embryos together with fluorescent microsphere-containing agarose columns in the cobweb holder. (**a**) Schematic of the cobweb holder mounting technique. The *Tribolium* embryo and an agarose column are mounted in vertical arrangement on a thin agarose film spanning a slotted hole. (**b**) Shown are transmission light and fluorescence microscopy images acquired in the DSLM of a mounted *Tribolium* embryo together with the associated agarose column. Red rectangles indicate the regions enlarged in the detail images, which depict a small area of the agarose column containing several fluorescent microspheres. Pink and teal arrowheads indicate two microspheres located approximately within the focal plane of the detection objective and thus within the illuminated light sheet plane. In the *z* maximum projection (ZM), additional microspheres located in front of or behind the focal plane become visible due to projection across the entire *z* stack. Scale bars, 100 µm (main images) and 20 µm (detail images).

Embryo collection and preparation were performed as described previously^28^. After collection, embryos were incubated at room temperature (23 °C ± 1 °C) for 15 h to reach the end of blastoderm formation. Embryos were mounted using the cobweb holder mounting technique^36,37^, and an agarose column (~460 µm in diameter) containing a 5% (v/v) suspension of fluorescent microspheres (1 µm diameter, T7282, Thermo Fisher Scientific) was positioned 50–150 µm below each embryo (Fig. 3a). In the DSLM, axial image (*z*) stacks of embryos were recorded together with a part of the fluorescent microspheres-containing agarose column (Fig. 3b) in a single fluorescence channel along four directions spaced 90° apart. Recording comprised up to 51 time points at 30 min intervals, corresponding to a total imaging duration of up to 25 h. Embryos and microspheres were illuminated with a laser power of 135 µW during a camera exposure time of 50 ms. After imaging, embryos were retrieved from the microscope chamber as described previously^28^.

### Image processing

**Fig. 4:**
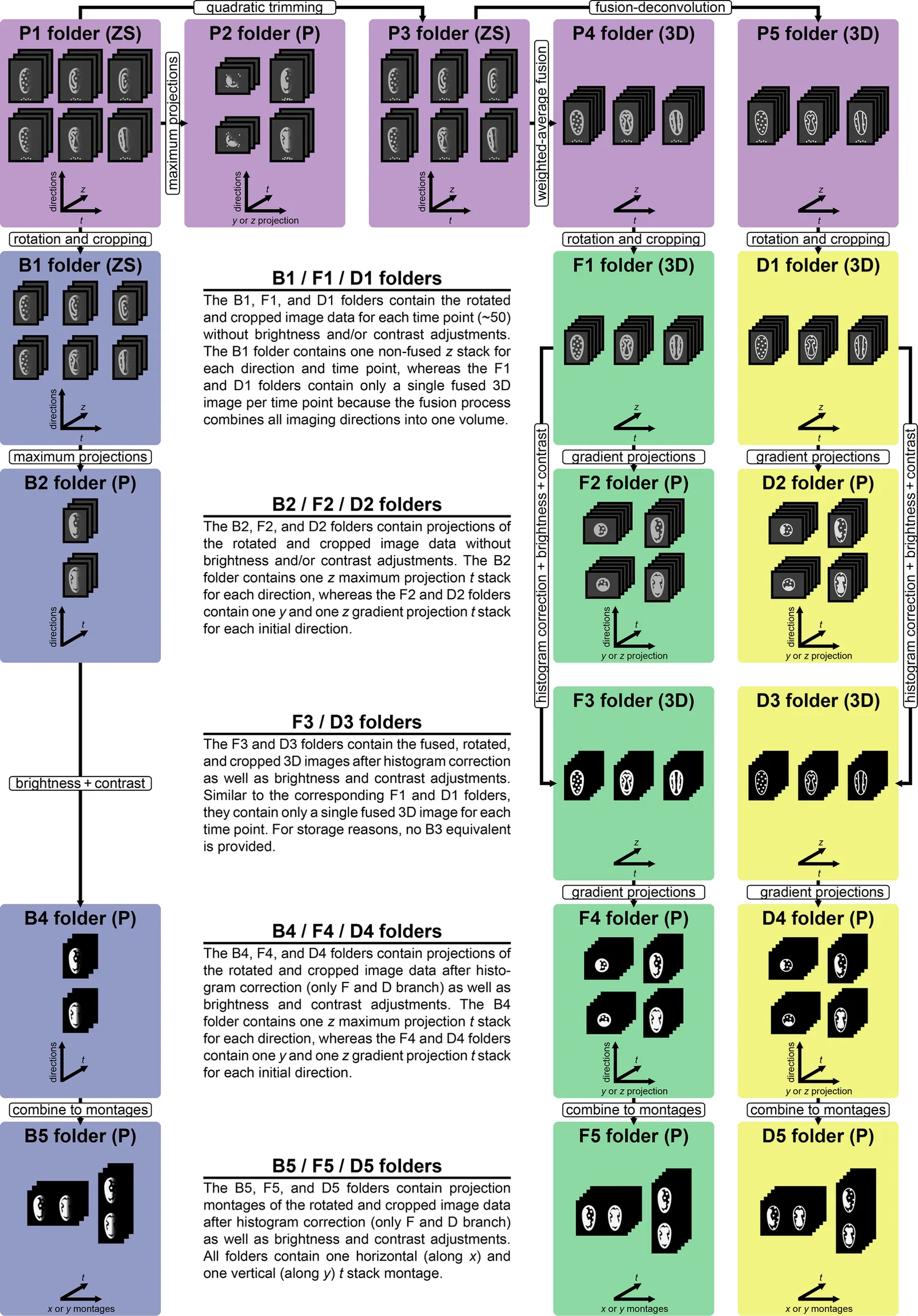
Image data processing workflow. The workflow comprised four processing branches: the preliminary processing branch (P, purple), the basic branch (B, blue), the weighted-average fusion branch (F, green), and the fusion-deconvolution branch (D, yellow). Rectangles represent folders within the dataset archives (cf. the Data Records section), whereas the corresponding headings indicate the type of files contained within each folder: *z* stacks (ZS), projections (P), or 3D images (3D). The B0 folder (not shown) contains metadata files.

All sixteen datasets (Table 1) were processed using an identical workflow (Fig. 4). Image processing started with the basic branch (denoted by ‘B’ in subfolder names), which comprises a simple processing routine for rapid visual inspection as described previously^26^, except that histogram matching was omitted. All subsequent image processing steps were performed exclusively in Fiji^38^ (based on ImageJ^39^ Version 1.53f).

**Table 1:** Overview of datasets and selected metadata.

DS	Total duration	Retrieval	Embryo rotation	Nuclei segmentation				Dataset access
				Uniform blastoderm		After window closure		
				TP	Nuclei number	TP	Nuclei number	
0001	24:00 h	developed into a healthy and fertile adult	~41° cw	—	—	0028	1094	part 1^42^, part 2^43^, part 3^44^
0002	24:00 h	developed into a healthy and fertile adult	~48° cw	0005	3557	0038	833	part 1^45^, part 2^46^, part 3^47^, part 4^48^, part 5^49^, part 6^50^
0003	23:00 h	developed into a healthy and fertile adult	~55° cw	—	—	0021	784	part 1^51^, part 2^52^, part 3^53^
0004	23:30 h	developed into an adult, no progeny	~15° ccw	0001	3637	—	—	part 1^54^, part 2^55^, part 3^56^
0005	24:00 h	developed into a healthy and fertile adult	~41° cw	0003	3507	0031	903	part 1^57^, part 2^58^, part 3^59^, part 4^60^, part 5^61^, part 6^62^
0006	23:00 h	developed into a healthy and fertile adult	~45° cw	0002	3457	0036	794	part 1^63^, part 2^64^, part 3^65^, part 4^66^, part 5^67^, part 6^68^
0007	25:00 h	developed into a healthy and fertile adult	~25° ccw	0007	3579	—	—	part 1^69^, part 2^70^, part 3^71^
0008	24:30 h	developed into a healthy and fertile adult	~53° cw	0006	3536	0034	681	part 1^72^, part 2^73^, part 3^74^, part 4^75^, part 5^76^, part 6^77^
0009	24:00 h	hatched but failed to reach the pupal stage	~0°	0016	3437	—	—	part 1^78^, part 2^79^, part 3^80^
0010	24:00 h	developed into an adult, no progeny	~5° ccw	0003	3653	—	—	part 1^81^, part 2^82^, part 3^83^
0011	24:00 h	developed into a healthy and fertile adult	~0°	0001	3702	—	—	part 1^84^, part 2^85^, part 3^86^
0012	24:30 h	developed into a healthy and fertile adult	~10° ccw	0002	3636	—	—	part 1^87^, part 2^88^, part 3^89^
0013	24:00 h	developed into a healthy and fertile adult	~0°	0009	3417	—	—	part 1^90^, part 2^91^, part 3^92^
0014	24:30 h	developed into a healthy and fertile adult	~27° ccw	0002	3649	—	—	part 1^93^, part 2^94^, part 3^95^
0015	24:30 h	developed into a healthy and fertile adult	~0°	0009	3609	—	—	part 1^96^, part 2^97^, part 3^98^
0016	24:30 h	developed into a healthy and fertile adult	~37° ccw	0001	3362	0022	826	part 1^99^, part 2^100^, part 3^101^, part 4^102^, part 5^103^, part 6^104^
Mean ± SD	—	—	—	—	3553 ± 104	—	845 ± 129	—

For convenient download, each dataset is divided into multiple parts. The first part contains all files from the basic branch, the second part contains all files from the weighted-average fusion and the fusion-deconvolution branches, the third part contains selected files from the preliminary branch, and the fourth to sixth parts (optional) contain the preliminary fusion *z* stacks. DS, dataset; TP, time point; cw, clockwise as seen from the anterior pole; ccw, counter-clockwise as viewed from the anterior pole; SD, standard deviation.

The preliminary processing branch (denoted by ‘P’ in subfolder names) comprises registration and fusion of *z* stacks acquired along multiple directions based on the multiview deconvolution plugin^40^ to obtain fused 3D images with roughly isotropic resolution and reduced shadowing artifacts. Firstly, based on the raw *z* stacks (P1 folder), *z* and *y* maximum projections were calculated for all *z* stacks and concatenated to time (*t*) stacks (P2 folder). Secondly, the *y* projection *t* stacks were used to estimate the mutual *x*-*z* quadratic footprint of the four *z* stacks recorded along different directions. Based on the estimate, the *z* stacks were trimmed accordingly, typically to sizes of 688–780 voxels along *x* and 172–195 voxels (i.e. planes) along *z* (P3 folder). Thirdly, for each direction, the 3D region containing the embryo was almost completely removed (i.e. blacked out) from the trimmed *z* stacks, except for a small cap at the top of the image volume (data not provided). These *ad hoc* stacks were used for registration, i.e. for calculating the transformation matrices based on interest points derived from (i) the fluorescent microsphere ‘cloud’ at the bottom of the image and (ii) a small number of nuclei within the apical cap at the top of the image that were visible in all four directions. Fourthly, the trimmed stacks were used together with the transformation matrices to calculate both preliminary weighted-average fusion-derived and fusion-deconvolution-derived 3D images. The point spread functions (PSFs) used for deconvolution were derived from the fluorescent microspheres, had dimensions of 23 × 23 × 23 voxels, and were used for deconvolution over ten iterations. Finally, the origin (coordinates 0-0-0) in the raw fused 3D images was reassigned to the left-top-front voxel in both fusion derivatives. In addition, the raw fusion-deconvolution-derived 3D images were converted from the resulting 32-bit floating point-based format to a 16-bit integer-based format (P4 and P5 folders).

**Fig. 5:**
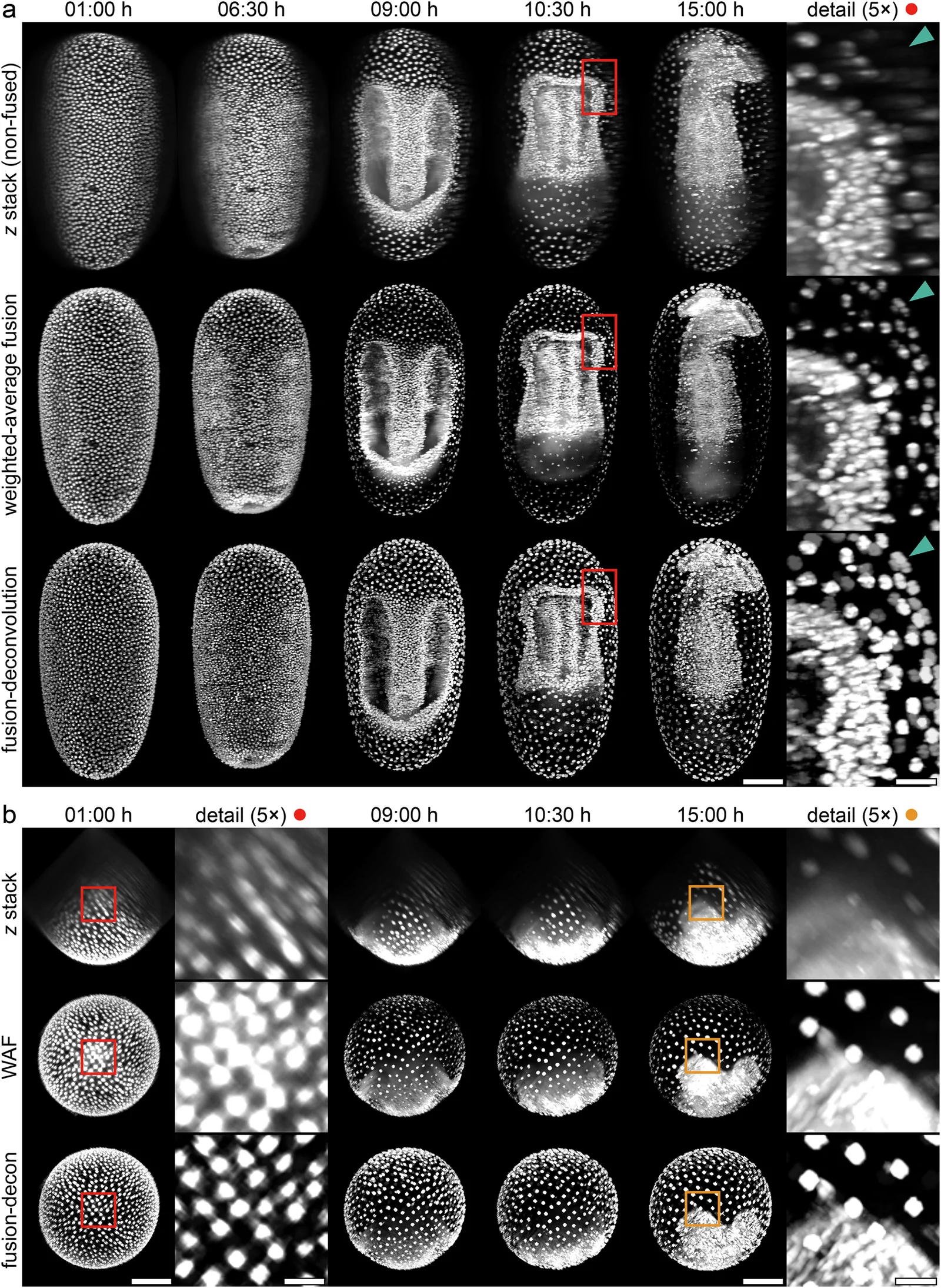
Comparison of non-fused *z* stacks with weighted-average fusion-derived and fusion-deconvolution-derived 3D images. (**a**) Juxtaposition of *z* projections of non-fused *z* stacks and the corresponding fused 3D images at five developmental time points. The embryo was initially recorded along four directions corresponding to two ventrolateral and two dorsolateral views, and one ventrolateral view was rotated around *y* to generate a ventral view (first row). The directional recordings were subsequently subjected to both weighted-average fusion and fusion-deconvolution and then rotated around all three spatial axes to align embryonic and image axes (second and third rows). Red rectangles indicate the regions enlarged in the detail images, which depict a small area in the anterior ventrolateral region. Serosa nuclei in the lateral region are clearly discernible only in the fused representations (teal arrowheads). (**b**) Juxtaposition of *y* projections of the non-fused *z* stacks and the corresponding fused 3D images at four developmental time points. Red and orange rectangles indicate the regions enlarged in the detail images, which depict a small area at the anterior pole. Serosa cell nuclei are only weakly recognizable in the non-fused projections due to shadowing artifacts. WAF, weighted-average fusion; fusion-decon, fusion-deconvolution. Image data are from dataset DS0005. Scale bars, 100 µm (main images) and 20 µm (detail images).

The resulting images were further processed in the weighted-average fusion branch (denoted by ‘F’ in subfolder names) and in the fusion-deconvolution branch (denoted by ‘D’ in subfolder names) to obtain rendering- and segmentation-ready 3D images (Fig. 5). Firstly, the fused images were rotated around all three spatial axes so that, at the time point immediately following serosa window closure (cf. Table 1, fifth column), the lateral axes of the embryos aligned with *x*, the anterior-posterior axes with *y* and the ventral-dorsal axes with *z*. Since the lateral and ventral-dorsal axes cannot be reliably identified at the uniform blastoderm stage, they cannot be physically aligned with the *x* and *z* axes of the microscope during mounting. Consequently, rotations around *y* reached up to 50°. In contrast, rotations around *x* and *z* were applied only to correct minor mounting-related misalignments with *y* and were small in magnitude (~3° on average for both axes). The 3D images were subsequently cropped to a size of 600 × 1000 × 600 voxels, or in the case of large embryos, 600 × 1100 × 600 voxels (F1 and D1 folders). Secondly, based on the axis-aligned and cropped 3D images, *y* and *z* gradient projections were calculated for four directions (in the orientations 0°, 90°, 180° and 270°). In these projections, front planes contribute more strongly to the final image than the rear planes. The resulting projections were concatenated into *y* and *z* projection *t* stacks (F2 and D2 folders). Thirdly, the axis-aligned and cropped 3D images were concatenated to *t*-*z* hyperstacks and subjected to histogram matching (Fiji: Bleach Correction → Histogram Matching) to equalize signal intensity over the entire time course. The hyperstacks were subsequently adjusted in brightness and contrast, and then split into individual 3D images again (F3 and D3 folders). Fourthly, background artifacts were removed by applying two 3D masks along the *x* and *z* axes, respectively (mask ROI (region-of-interest) files are provided in the B0 folder). Fifthly, based on the axis-aligned, cropped, and intensity-adjusted 3D images, *y* and *z* gradient projections were calculated for four directions (in the orientations 0°, 90°, 180° and 270°) and concatenated into *t* stacks (F4 and D4 folders). Finally, the *t* stacks for all directions were combined into horizontal and vertical *t* stack montages (F5 and D5 folders).

### Dataset staging

Datasets were manually staged based on five morphologically characteristic developmental landmarks: (i) uniform blastoderm (shortly before the onset of rearrangement), (ii) posterior plate or posterior pit at the retracted posterior pole, (iii) horseshoe-shaped amniotic fold at the ventral side, (iv) serosa window clearly visible at the ventral side, and (v) onset of germband elongation (shortly after serosa window closure). Except for two datasets (DS0001 and DS0003), in which imaging commenced after the embryos had progressed beyond the uniform blastoderm stage, all stages could be reliably identified across all datasets (a dataset-resolved overview is provided in Supplementary Fig. 1). No temporal alignment, registration, normalization, or time-warping procedures were applied across datasets. Accordingly, identical time points in different datasets do not necessarily correspond to identical developmental stages.

### Nuclear segmentation and spatial quantification

**Fig. 6:**
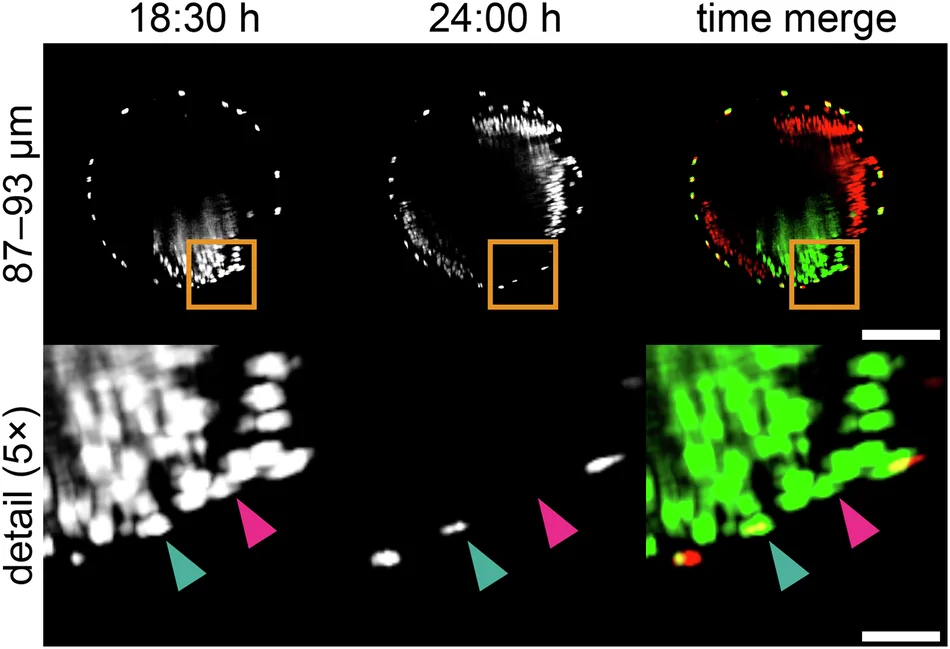
Identification of serosa nuclei in close proximity to the developing germband using ‘time-merged’ 3D images. Shown are partial (~7 µm) *y* projections of a ‘time-merged’ 3D image. Between the stage immediately following serosa window closure (first column; green in third column) and the final time point of the dataset (second column; red in third column), the germband of the embryo shown here rotated by ~48° clockwise (as viewed from the anterior pole) around the anterior-posterior axis (i.e. *y*) within the serosa. This rotation enables identification of serosa nuclei in the time-merged volume: nuclei belonging to the serosa retain signal at the same spatial position across both time points, whereas nuclei belonging to the amnion/germband do not (third column). Orange rectangles indicate the regions enlarged in the detail images, which depict a small area in the ventrolateral region where the serosa and germband were in contact at 18:30 h but separated at 24:00 h. Based on the presence or absence of overlapping signal across the two time points, the nucleus marked by the teal arrowhead was classified as belonging to the serosa, whereas the nucleus marked by the pink arrowhead was not. Image data are from dataset DS0002. Scale bars, 100 µm (main images) and 20 µm (detail images).

Blastodermal nuclei at the uniform blastoderm stage as well as serosa nuclei after serosa window closure were segmented in the axis-aligned, cropped, and intensity-adjusted 3D images obtained at the end of the fusion-deconvolution branch (D3 folder). Segmentation was performed manually in the dedicated 3D image visualization and analysis software Arivis Pro (version 4.2.1, Zeiss Microscopy) using the ‘Magic Wand’ tool in the 2D viewer mode (search tolerance 2–10%, holes not allowed). Since some serosa nuclei at the onset of germband elongation were difficult to demarcate due to their proximity to the developing germband, segmentation was restricted to datasets in which the germband rotated by ≥ 35° around the anterior-posterior axis after serosa window closure (cf. Table 1, fourth column). In these datasets, serosa nuclei were segmented using ‘time-merged’ 3D images displaying (i) the time point of interest in green and (ii) the final time point of the respective time series in red. This representation facilitated identification of serosa nuclei based on their spatial correspondence across time points (Fig. 6). In three datasets (DS0003, DS0006, and DS0016), nuclei identification in specific regions was hindered by suboptimal local contrast in the standard intensity-adjusted 3D images. To address this issue, additional 3D ‘helper’ images with time point-specific brightness and contrast adjustments were generated to support accurate segmentation.

## Data Records

The SLICE-2 datasets are available for download at Zenodo as ZIP-compressed TIFF files and can be accessed either through the dataset collection overview page^41^ or directly via the individual digital object identifiers^42–104^. For each dataset, files corresponding to the different image processing branches are distributed across multiple ZIP archives (Table 1). The first archive contains all files from the basic branch (part 1, ‘B’), the second archive contains all files from the weighted-average fusion and fusion-deconvolution branches (part 2, ‘F’ and ‘D’), and the third archive contains selected files from the preliminary branch (part 3, ‘P1’–‘P3’). For the five datasets in which both blastodermal and serosa nuclei were segmented, additional archives are provided. The fourth archive contains the preliminary weighted-average fusion-derived 3D images (part 4, ‘P4’), whereas the fifth and sixth archives contain the preliminary fusion-deconvolution-derived 3D images (parts 5 and 6, ‘P5’). For the remaining eleven datasets, these images can be reproduced from the image data in the P3 folder in combination with the fusion metadata stored in the B0 folder. An overview of the folder structure is provided in Table 2. Each entry also includes comprehensive experimental metadata in the form of a human- and machine-readable XLSX file. In addition, the first entry for each dataset also contains intensity-adjusted *z* projection *t* stack montage TIFF files together with corresponding AVI files for rapid inspection of the respective dataset in Fiji or any standard movie player. The complete collection has a total size of ~1.65 Terabytes.

**Table 2:** Overview of the data folder structure across the image processing branches.

Branch	Folder	RelVol	Rationale
P branch	(P1)-ZStacks-RS	+ + + +	Contains raw *z* stacks (‘RS’). Feeds into the B branch. **This folder contains the top-level data in the processing hierarchy**.
	(P2)-TStacks-YR-ZR	+	Contains *t* stacks (‘TS’) of *y* and *z* maximum projections derived from the raw *z* stacks (‘YR’ and ‘ZR’, respectively).
	(P3)-QStacks	+ + +	Contains *z* stacks cropped to mutual quadratic footprints (‘QS’).
	(P4)-QStacks-Fusion	+ + +	Contains the preliminary weighted-average fusion-derived 3D images. Data only provided for the five datasets in which both blastodermal and serosa nuclei were segmented. Feeds into the F branch.
	(P5)-QStacks-Decon	+ + + +	Contains the preliminary fusion-deconvolution-derived 3D images. Data only provided for the five datasets in which both blastodermal and serosa nuclei were segmented. Feeds into the D branch.
B branch	(B0)-Metadata	-	Contains fusion metadata. The comprehensive dataset metadata XLSX file is normally stored in this folder but has been extracted to facilitate independent access without downloading the full dataset.
	(B1)-ZStacks-ZS	++	Derives from the (P1) folder. Contains non-fused, rotated, and cropped *z* stacks (‘ZS’). *The folder contains subfolder layers for channels and directions*.
	(B2)-TStacks-ZM	+	Contains *t* stacks (‘TS’) of *z* maximum projections (‘ZM’) derived from the non-fused, rotated, and cropped *z* stacks. *The folder contains subfolders for channels and directions*.
	(B4)-TStacks-ZN	+	Contains *t* stacks (‘TS’) of *z* maximum projections (‘ZA’) derived from the non-fused, rotated, and cropped *z* stacks that were subjected to intensity adjustment. *The folder contains subfolders for channels and directions*.
	(B5)-TStacks-ZN-Montage	+	Contains horizontal and vertical direction montages (‘AX’ and ‘AY’, respectively) of *t* stacks (‘TS’) derived from the *z* maximum projections (‘ZA’) of non-fused, rotated, and cropped *z* stacks that were subjected to intensity adjustment. **The montages are intended for quick visual inspection of the respective dataset/processing branch**.
F branch	(F1)-ZStacks-Fusion-ZS	++	Derives from the (P4) folder. Contains weighted-average fusion-derived, rotated, and cropped 3D images (‘ZS’). *The folder contains two subfolder layers for channels and directions*.
	(F2)-TStacks-Fusion-YM-ZM	+	Contains *t* stacks (‘TS’) of *y* and *z* gradient projections (‘YM’ and ‘ZM’, respectively) derived from the weighted-average fusion-derived, rotated, and cropped 3D images. *The folder contains two subfolder layers for channels and directions*.
	(F3)-ZStacks-Fusion-NS	+	Contains weighted-average fusion-derived, rotated, and cropped *z* stacks (‘NS’) that were subjected to histogram matching and intensity adjustment. *The folder contains two subfolder layers for channels and directions*.
	(F4)-TStacks-Fusion-YN-ZN	+	Contains *t* stacks (‘TS’) of *y* and *z* gradient projections (‘YH’ and ‘ZH’, respectively) derived from the weighted-average fusion-derived, rotated, and cropped 3D images that were subjected to histogram matching and intensity adjustment. *The folder contains two subfolder layers for channels and directions*.
	(F5)-TStacks-Fusion-ZN-Montage	+	Contains horizontal and vertical direction montages (‘AX’ and ‘AY’, respectively) of *t* stacks (‘TS’) derived from the *z* gradient projections (‘ZH’) of weighted-average fusion-derived, rotated, and cropped *z* stacks that were subjected to histogram matching and intensity adjustment. **The montages are intended for quick visual inspection of the respective dataset/processing branch**.
D branch	(D1)-ZStacks-Decon-ZS	+++	Derives from the (P5) folder. Contains fusion-deconvolution-derived, rotated, and cropped 3D images (‘ZS’). *The folder contains two subfolder layers for channels and directions*.
	(D2)-TStacks-Decon-YM-ZM	+	Contains *t* stacks (‘TS’) of *y* and *z* gradient projections (‘YM’ and ‘ZM’, respectively) derived from the fusion-deconvolution-derived, rotated, and cropped 3D images. *The folder contains two subfolder layers for channels and directions*.
	(D3)-ZStacks-Decon-NS	+	Contains fusion-deconvolution-derived, rotated, and cropped *z* stacks (‘NS’) that were subjected to histogram matching and intensity adjustment. **Segmentation data derive from the files in this folder**. *The folder contains two subfolder layers for channels and directions*.
	(D4)-TStacks-Decon-YN-ZN	+	Contains *t* stacks (‘TS’) of *y* and *z* gradient projections (‘YH’ and ‘ZH’, respectively) derived from the fusion-deconvolution-derived, rotated, and cropped 3D images that were subjected to histogram matching and intensity adjustment. *The folder contains two subfolder layers for channels and directions*.
	(D5)-TStacks-Decon-ZN-Montage	+	Contains horizontal and vertical direction montages (‘AX’ and ‘AY’, respectively) of *t* stacks (‘TS’) derived from the *z* gradient projections (‘ZH’) of fusion-deconvolution-derived, rotated, and cropped *z* stacks that were subjected to histogram matching and intensity adjustment. **The montages are intended for quick visual inspection of the respective dataset/processing branch**.

The folders are organized into four branches: the preliminary processing branch (P), the basic branch (B), the weighted-average fusion branch (F), and the fusion-deconvolution branch (D). In the folder names, ‘N’ serves as a placeholder indicating the type of intensity adjustment applied: either only intensity adjustment (abbreviated as ‘AS’ in *z* stacks file names and ‘ZA’ in *z* maximum projection file names), or histogram correction followed by intensity adjustment (abbreviated as ‘HS’ in *z* stacks file names, ‘YH’ in *y* gradient projection file names, and ‘ZH’ in *z* gradient projection file names). Bold formatting highlights quick-start information, whereas italics denote explanatory details related to the subfolder hierarchy. RelVol, approximate estimate of relative data volume within a dataset.

Of the sixteen datasets, blastodermal nuclei were segmented in fourteen, excluding the two datasets in which imaging commenced after the embryos had progressed beyond the uniform blastoderm stage. Serosa nuclei were segmented in the seven datasets in which the germband underwent sufficient rotation around the anterior-posterior axis (*y*). The segmentation data are available for download at Zenodo^105^ as ZIP archives containing (i) copies of the axis-aligned, cropped, and intensity-adjusted 3D images from the fusion-deconvolution branch (including the time-merged 3D images and ‘helper’ images, where applicable) used for segmentation (Table 1, fifth column), (ii) the corresponding segmentation SIS files together with their associated resources, and (iii) machine-readable XLSX files containing nuclei coordinates based on the intensity-weighted center of mass and sorted according to the center of the bounding box, first by *y* (primary), then *x* (secondary), and finally *z* (tertiary). The segmentation data have a total size of ~2 Gigabytes.

## Technical Validation

Throughout the entire project and during each experiment, particular emphasis was placed on ensuring dataset quality, homogeneity, comparability, and reusability. Key technical considerations included:

### Homozygous imaging cultures

After completion of the mating procedure associated with the AGOC vector concept^106^, the AGOC{ATub’H2A/H2B°NB-mEmerald} #1 transgenic subline carried a single homozygous transgene insertion^26^. Consequently, the corresponding imaging culture produced exclusively homozygous embryos, eliminating variation in fluorescence signal intensity and/or pattern that may otherwise arise from differences in transgene dosage and/or multiple genomic insertions.

### Microscope calibration and effective laser power

Prior to each recording, the DSLM was calibrated using a two-step routine^36^ to prevent common issues such as offsets or tilts between the light sheet and the focal plane of the detection objective. The effective laser power, defined as the power at the location of the embryos during imaging rather than the nominal output of the laser module, was monitored regularly. When deviations were detected, the laser output was adjusted to maintain consistent illumination intensity across all dataset acquisitions.

### Mounting arrangement

Since embryos and agarose columns containing the fluorescent microspheres were positioned along *y* with sufficient separation between them, the illumination and detection light paths to the embryos were not obstructed by the microspheres. Consequently, in all but one dataset (DS0002), the fluorescent microsphere signal could be completely removed from the *z* stacks and 3D images by simple horizontal cropping, eliminating the need for additional masking or specialized processing.

### Validation of non-invasive imaging

To verify that the imaging procedure, including light exposure, did not induce developmental defects, all embryos were retrieved after recording and reared, and the resulting adults were assessed for fertility as described previously^28^. All embryos hatched morphologically intact. Of the resulting larvae, all but one (DS0009) developed into healthy adults. Among the resulting adults, fertility was confirmed for all but two individuals (DS0004 and DS0010).

### Registration quality

**Fig. 7:**
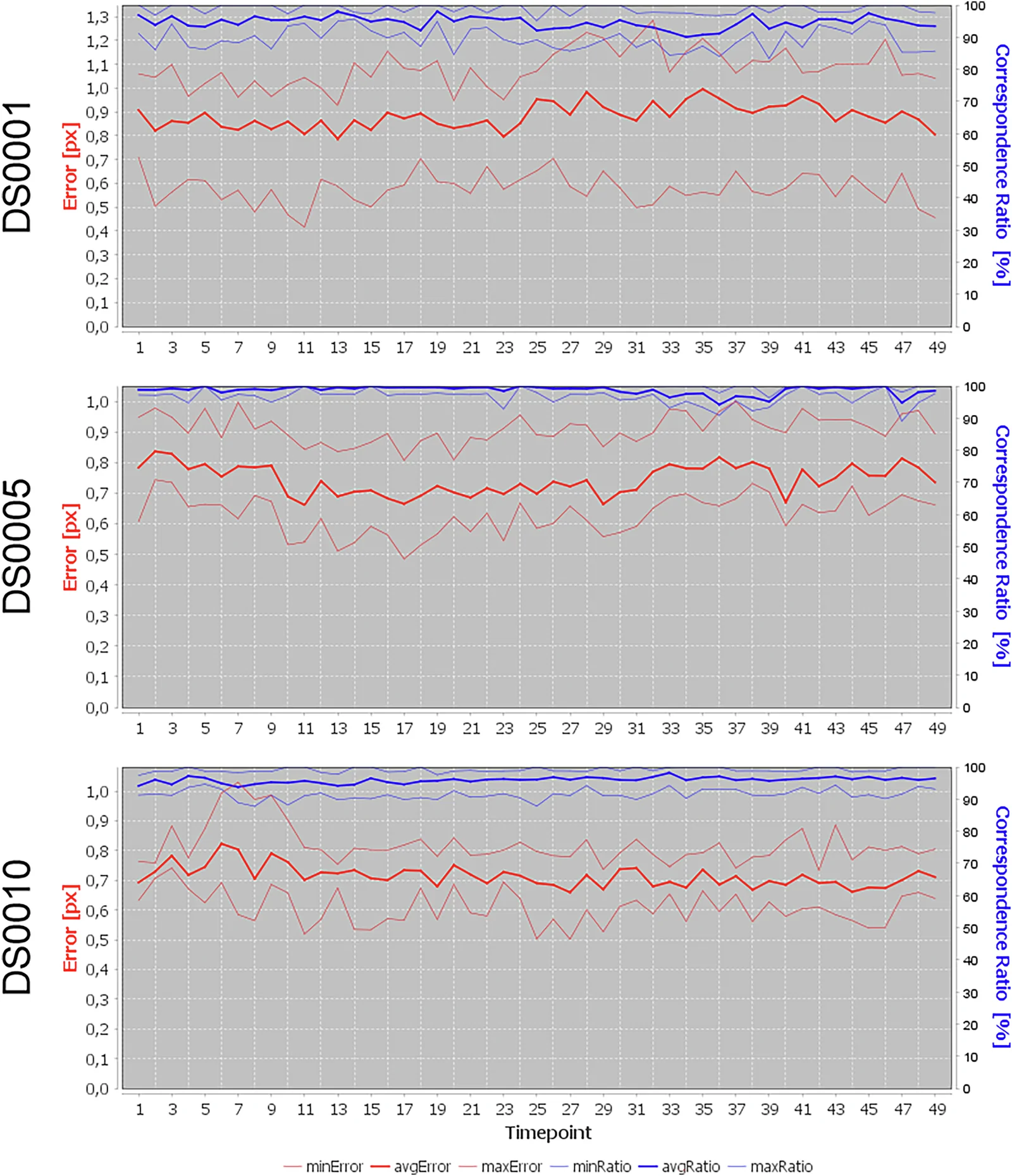
Assessment of registration quality. The graphs summarize multiview registration performance across the entire acquisition period for three representative datasets. Shown are the minimum, average, and maximum registration errors in pixels (minError, avgError, maxError; red curves, left axis) and the minimum, average, and maximum correspondence ratios (minRatio, avgRatio, maxRatio; blue curves, right axis) computed across the six direction pairs (1 ↔ 2, 1 ↔ 3, 1 ↔ 4, 2 ↔ 3, 2 ↔ 4, and 3 ↔ 4). The correspondence ratios indicate the fraction of identified fluorescent microspheres that were successfully matched across the four acquisition directions, i.e. the fraction of correspondences established by the registration algorithm. Across all three datasets, the average registration error remains below 1 pixel, which is small relative to the final dimensions of the fused 3D images (i.e. 600 × 1000 × 600 voxels along *x*, *y*, and *z*, respectively). At the same time, the mean correspondence ratios consistently exceeded 90%, indicating that the vast majority of fluorescent microspheres were successfully matched across all direction pairs. Together, these metrics demonstrate that the multiview registration procedure is reliable and robust, providing a strong foundation for high-quality fusion and downstream quantitative analyses.

Registration quality was evaluated for each dataset to ensure accurate alignment of *z* stacks acquired along multiple directions. Two quantitative metrics were assessed: residual registration error and interest-point correspondence ratio. Across all datasets, the residual registration error typically remained below one pixel, indicating high spatial fidelity of the registration procedure. The correspondence ratio consistently exceeded 90%, reflecting robust identification and alignment of landmarks (i.e. fluorescent microspheres) across *z* stacks acquired along multiple directions (Fig. 7).

### Validation of image registration and fusion

**Fig. 8:**
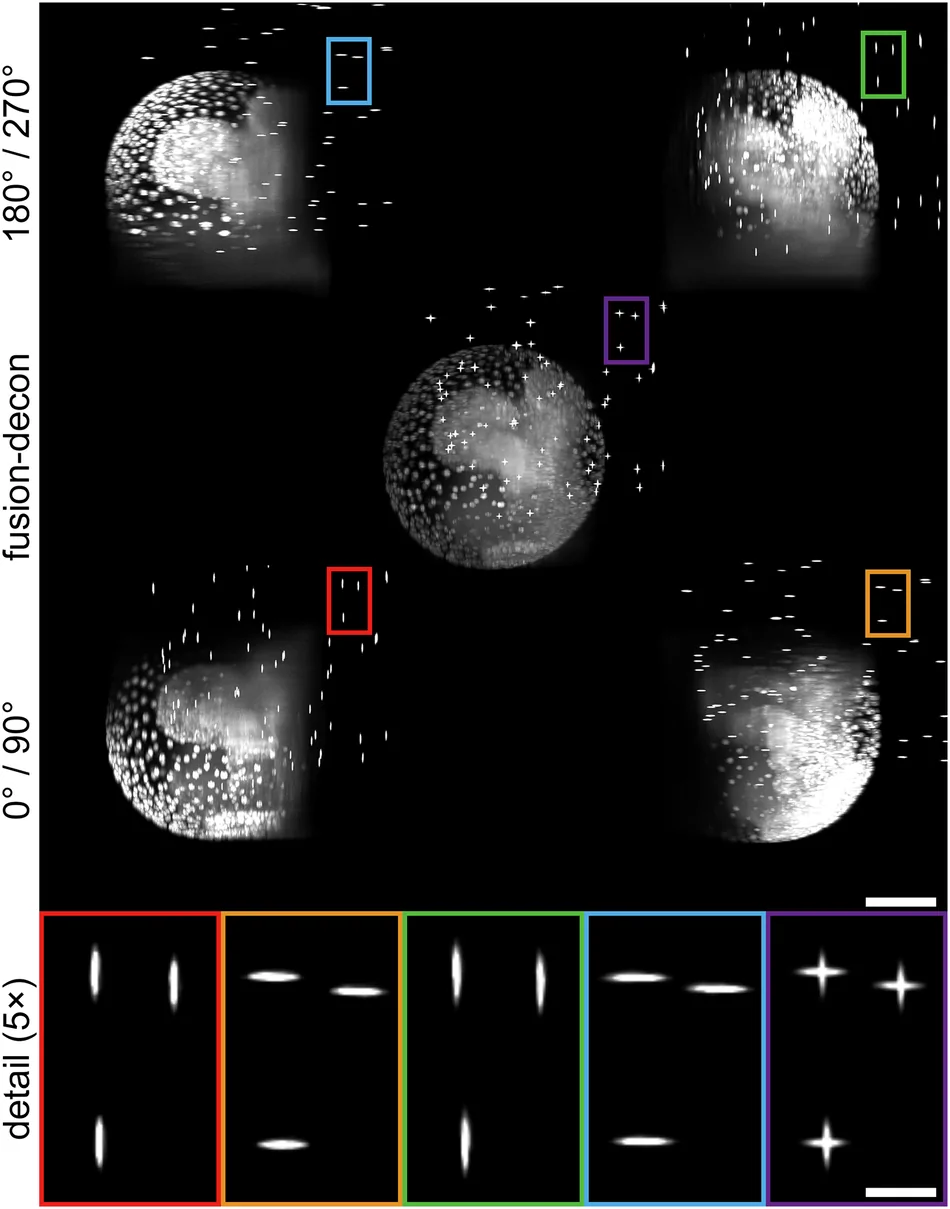
Fluorescent microsphere-based validation of registration and fusion. Shown are *y* projections of *ad hoc z* stacks trimmed to mutual quadratic footprints (outer panels) and the corresponding fused 3D image prior to rotation and cropping, i.e. with the fluorescent microsphere ‘cloud’ still present (inner panel). All five detail images display the same three fluorescent microspheres. The compass-star-like geometry of the signal in the detail image derived from the fused 3D image indicates correct registration and fusion. Notably, the projection derived from the fused 3D image exhibits a smaller overall point spread than the individual *ad hoc* projections, reflecting the effects of fusion and deconvolution. Fusion-decon, fusion-deconvolution. Image data are from dataset DS0005. Scale bars, 100 µm (main images) and 20 µm (detail images).

**Fig. 9:**
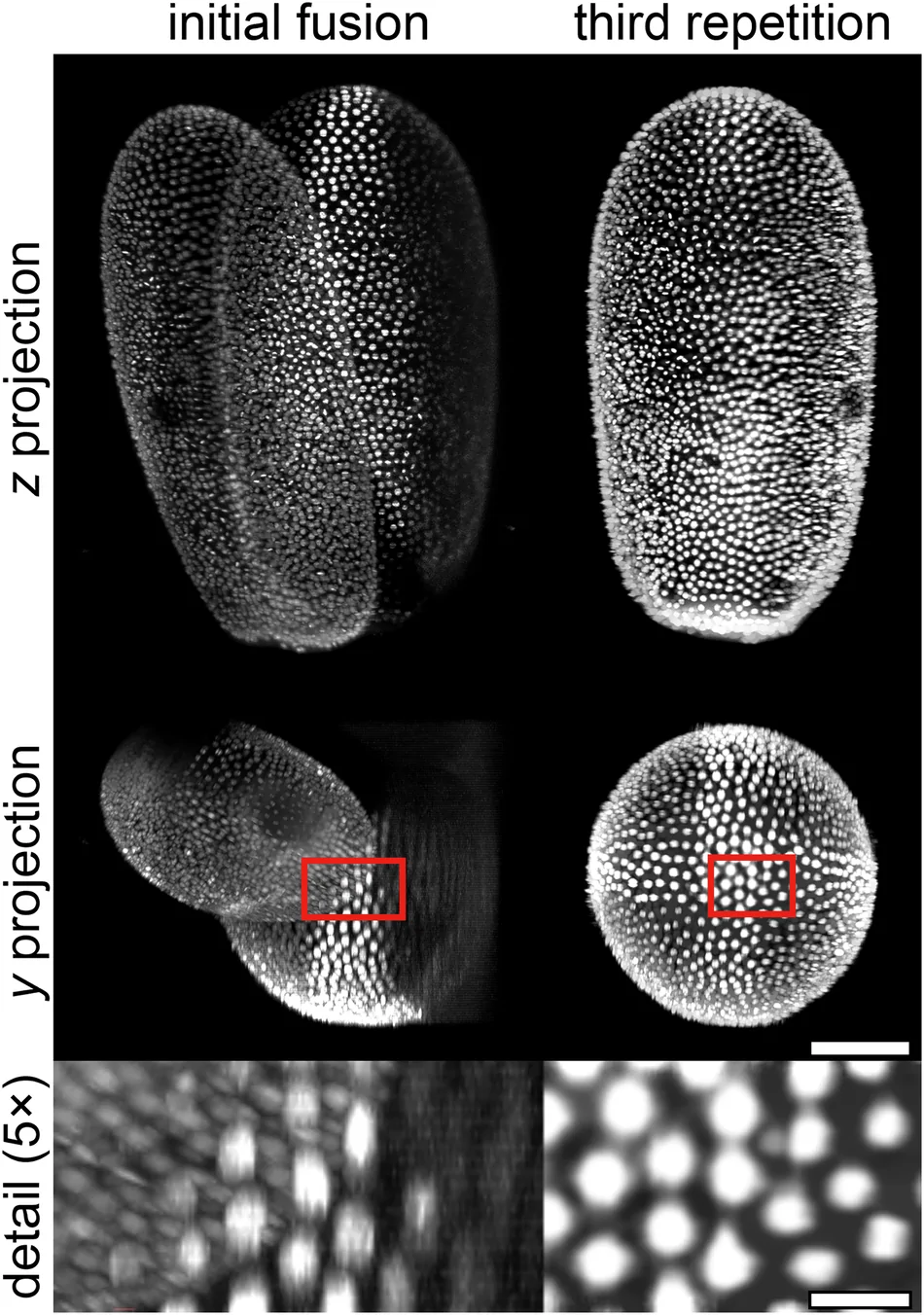
Fusion error. Shown are *z* and *y* projections of weighted-average fusion-derived 3D images. At the 13^th^ time point (TP0013), multiple *z* stacks were incorrectly positioned during the fusion process. After two additional fusion attempts with slightly altered parameters produced similar errors, fusion was repeated using the registration parameters from the subsequent time point. This was the only fusion error observed across all time points of the sixteen datasets. Red rectangles indicate the regions enlarged in the detail images, which depict a small area at the anterior pole. In the initial fusion, the nuclei located directly at the pole were likely reconstructed primarily from a single acquisition direction. Image data are from dataset DS0010. Scale bars, 100 µm (main images) and 20 µm (detail images).

Correct image registration and fusion were verified through manual visual inspection of *ad hoc* projections of the raw fused 3D images that still retained fluorescent microsphere signal. Particular attention was paid to the microsphere-derived signal, which appears compass-star-shaped in *y* projections of correctly fused reconstructions (Fig. 8). Across a total of 786 independent fusion processes, only a single fusion error was identified (DS0010, TP0013). In this occurrence, the four *z* stacks were incorrectly fused during the initial run (Fig. 9) as well as in two subsequent repetitions. As a workaround, fusion was performed using the transformation matrices from the subsequent time point. This incident is documented in the corresponding metadata.

### Estimation of effective spatial resolution

**Fig. 10:**
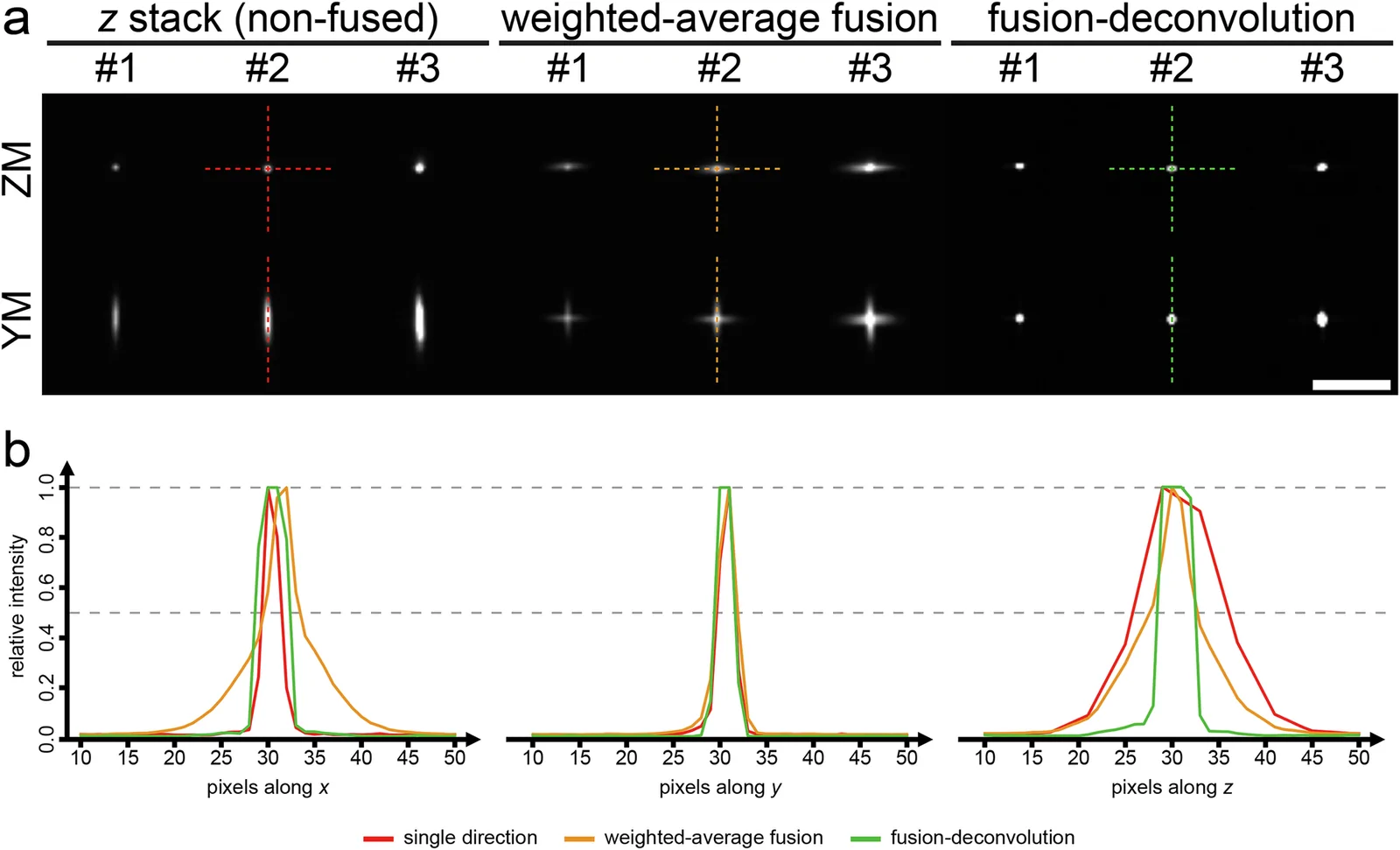
Estimation of effective spatial resolution. (**a**) Shown are *z* and *y* projections (ZM and YM, respectively) of three fluorescent microspheres differing in fluorescence intensity (weak, #1; intermediate, #2; strong, #3). Microspheres were extracted from a non-fused *z* stack with interplane interpolation (P3 folder) as well as from the corresponding preliminary weighted-average fusion-derived and fusion-deconvolution-derived 3D images (P4 and P5 folders), respectively, of the image processing workflow. Dashed lines indicate the directions along which the relative intensity profiles shown in (**b**) were measured. Scale bar, 20 µm. (**b**) Relative fluorescence intensity profiles of microsphere #2 measured along the *x*, *y*, and *z* axes. Note that certain intensity values in the fusion-deconvolution-derived 3D image reached the 16-bit saturation limit (2^16^), causing the corresponding profile peaks to appear partially flattened. Image data are from dataset DS0005.

Effective spatial resolution was estimated by analyzing the fluorescent microspheres used for image registration. Specifically, fluorescent microspheres of varying intensity were cropped in 3D from (i) trimmed *z* stacks, (ii) preliminary weighted-average fusion-derived 3D images, and (iii) preliminary fusion-deconvolution-derived 3D images (Fig. 10a). Following local background subtraction, intensity profiles through the centers of the microspheres were extracted along *x*, *y*, and *z* (Fig. 10b), and the full width at half maximum (FWHM) was determined for each axis (Table 3). These measurements indicate that the initially pronounced anisotropy between lateral and axial resolution (~1:5) was substantially reduced following multiview fusion and deconvolution, resulting in a more isotropic effective resolution. It should be noted, however, that the fluorescent microspheres had a nominal diameter of 1 µm and were imaged at an effective lateral voxel spacing of 0.645 µm. Consequently, the measured FWHM values are influenced both by the finite sphere diameter and by discrete image sampling and should therefore be interpreted only as empirical approximations of the effective spatial resolution.

**Table 3:** Estimation of effective spatial resolution.

Image	Folder	FWHM along *x*	FWHM along *y*	lateral FWHM	FWHM along *z*
trimmed *z* stacks	P3	1.38 µm ± 0.11 µm	1.32 µm ± 0.14 µm	1.35 µm ± 0.12 µm	6.83 µm ± 0.23 µm
preliminary weighted-average fusion-derived 3D image	P4	2.52 µm ± 0.09 µm	1.52 µm ± 0.05 µm	2.02 µm ± 0.07 µm	3.27 µm ± 0.68 µm
preliminary fusion-deconvolution-derived 3D image	P5	2.39 µm ± 0.33 µm	1.61 µm ± 0.33 µm	2.00 µm ± 0.32 µm	2.91 µm ± 0.92 µm

Estimates of both lateral (*x* and *y*) and axial (*z*) resolution are based on full width at half maximum (FWHM) measurements of identical fluorescent microspheres in (i) trimmed *z* stacks with interplane interpolation, (ii) preliminary weighted-average fusion-derived 3D images, and (iii) preliminary fusion-deconvolution-derived 3D images. Lateral FWHM values represent the arithmetic mean of the corresponding measurements along *x* and *y*. Values are given in micrometers ± standard deviation (n = 3). One pixel corresponds to 0.645 µm (cf. the Light sheet fluorescence microscopy subsection in the Methods section).

### Assessment of fluorescence intensity uniformity

**Fig. 11:**
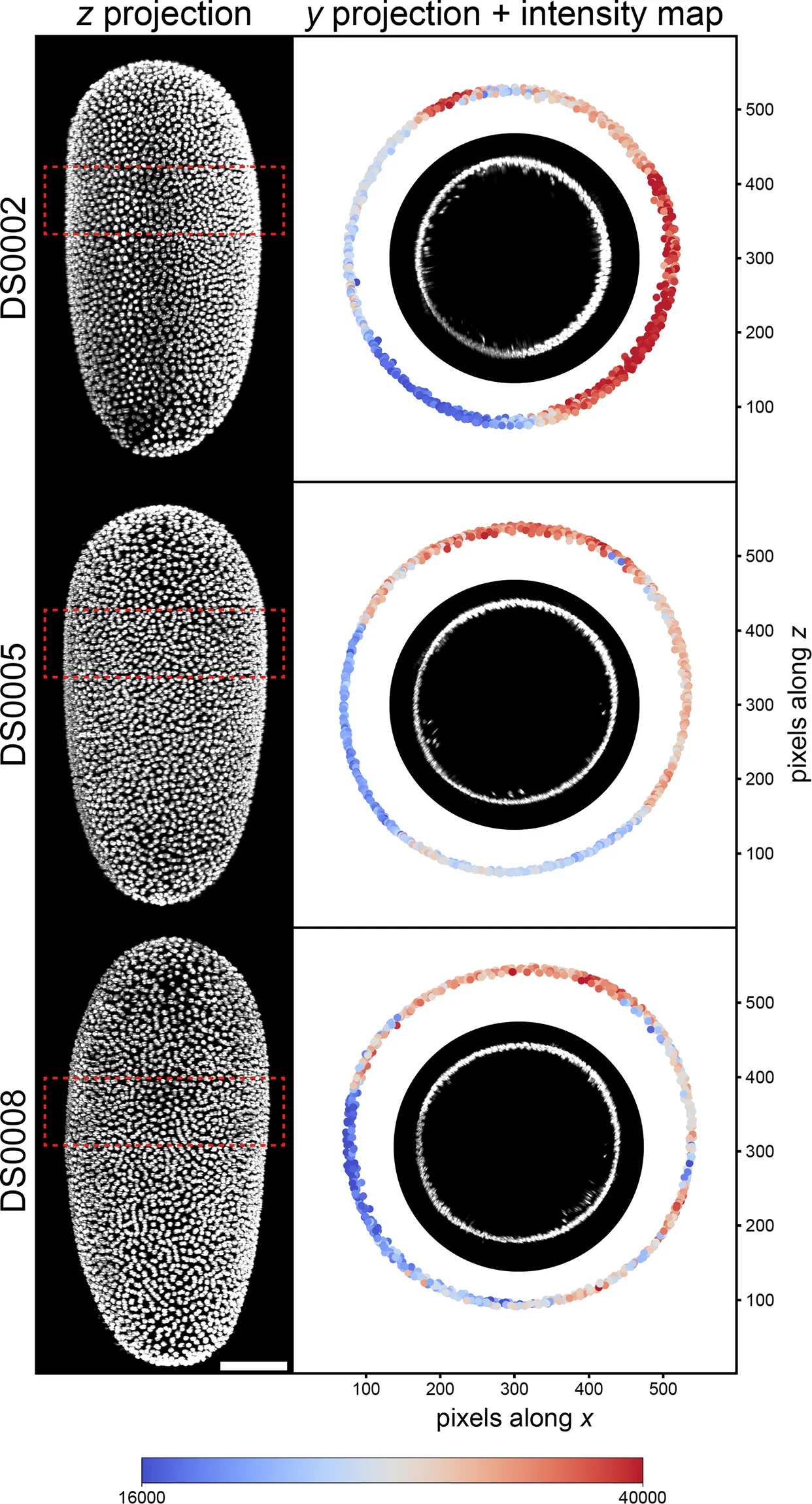
Quantification of nuclear fluorescence intensity distributions across transverse (*x*-*z*) subregions of three *Tribolium* embryos at the uniform blastoderm stage. The analyses were based on segmented nuclear masks. Red dashed rectangles in the *z* projections indicate the regions used to calculate the partial *y* projections and to select nuclei included in the corresponding *x*-*z* fluorescence intensity maps. Colored dots represent segmented nuclei positioned according to their *x*-*z* coordinates and color-coded by relative mean fluorescence intensities in arbitrary units within the 16-bit intensity range. All three embryos exhibit a moderate degree of spatial fluorescence intensity heterogeneity. Scale bar, 100 µm.

As an additional quality control, spatial distributions of nuclear fluorescence intensities were quantified across transverse subregions of embryos at the uniform blastoderm stage, which is particularly well suited for this type of assessment because of the comparatively homogeneous blastoderm morphology. The analysis revealed a moderate degree of spatial fluorescence intensity heterogeneity (Fig. 11), likely attributable to the cobweb holder mounting technique, as illumination and/or detection light must traverse the agarose film in some of the four imaging directions. Despite this heterogeneity, local signal-to-noise ratios remained sufficiently high to permit robust nuclear segmentation without noticeable impairment. Nevertheless, the observed intensity variation should be taken into account in sensitive downstream analyses and may, where necessary, require appropriate normalization or compensation strategies.

### Segmentation validation by a second researcher

Manual segmentation of blastodermal nuclei at the uniform blastoderm stage as well as serosa nuclei after serosa window closure was performed by two researchers to ensure accuracy. Following the initial segmentation by the first researcher, all segmented nuclei were systematically reviewed by a second researcher. The review was performed using both the 2D viewer and the 3D viewer modes in Arivis Pro. This double-checking procedure was implemented to minimize observer bias and segmentation errors. To quantify segmentation fidelity, the number of corrections made by the second reviewer across seven datasets at the uniform blastoderm stage (DS0004, DS0007, DS0009, DS0011, DS0012, DS0013, and DS0014) was documented. This yielded 5.1 ± 4.3 false negatives and 7.7 ± 3.8 false positives (mean ± standard deviation, ~0.14% and ~0.22%, respectively, of the mean total nuclei number across these datasets).

### Example of biologically informative quantitative analyses

**Fig. 12:**
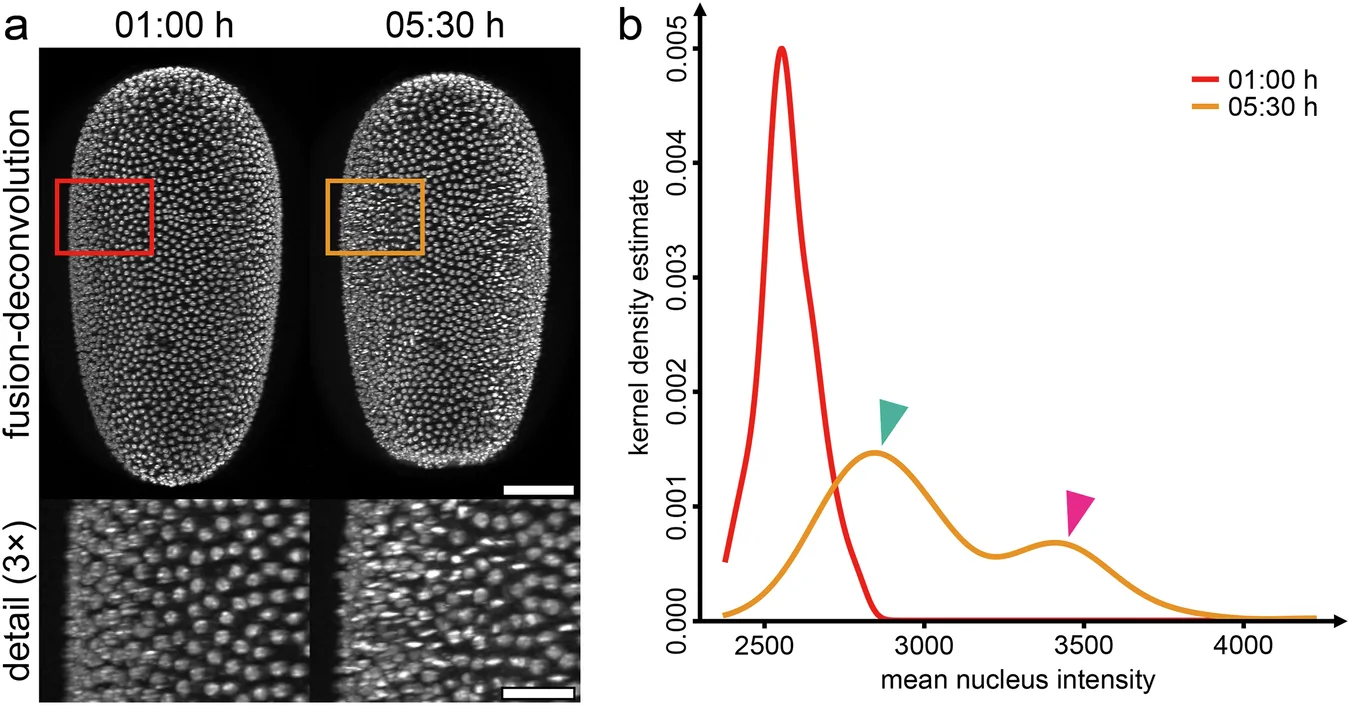
Identification of cell-cycle states as an example of biologically informative quantitative analyses. (**a**) Shown are *z* projections of fusion-deconvolution-derived 3D images at two developmental time points. At 01:00 h, the embryo is at the uniform blastoderm stage, whereas at 05:30 h, it is at the onset of gastrulation. Red and orange rectangles indicate the regions enlarged in the detail images, which depict a small area in the anterior ventrolateral region of the embryo. At 01:00 h, nuclei predominantly exhibit spherical morphology characteristic of interphase nuclei (G1, S and G2 phases of the cell cycle). At 05:30 h, numerous metaphase plates are visible, indicating that multiple cells are undergoing mitosis (M phase). Image data are from dataset DS0005. Scale bars, 100 µm (main image) and 30 µm (detail image). **(b)** Kernel density estimations based on mean nuclear fluorescence intensity values for segmented nuclear masks located within the regions outlined in (**a**), comprising 114 and 188 masks for the 01:00 h and 05:30 h time points, respectively. At 01:00 h, the intensity distribution is predominantly unimodal, whereas at 05:30 h, a bimodal distribution is observed. The left peak (teal arrowhead) corresponds primarily to nuclei in G1, S, and G2 phases, whereas the right peak (pink arrowhead) corresponds predominantly to mitotic nuclei in M phase.

To demonstrate the potential for extraction of biologically relevant information from the imaging data, nuclear fluorescence intensities were quantified at two developmental stages: the uniform blastoderm stage, during which mitotic nuclei are largely absent, and the onset of gastrulation, during which certain cells of the condensing embryonic rudiment undergo mitosis. These measurements were used to infer the approximate distribution of cells across different cell-cycle phases based on differences in chromatin condensation state (Fig. 12). These observations indicate that the datasets support biologically informative quantitative analyses at single-cell resolution beyond the spatial quantification presented in this data descriptor.

## Usage Notes

The image data structure (cf. the Data Records section) is compatible with any software capable of handling dynamic 3D image data. Fiji^38^, an open-source framework widely used for handling multi-dimensional microscopy data, is recommended as the primary platform for image processing and analysis.

**Fig. 13:**
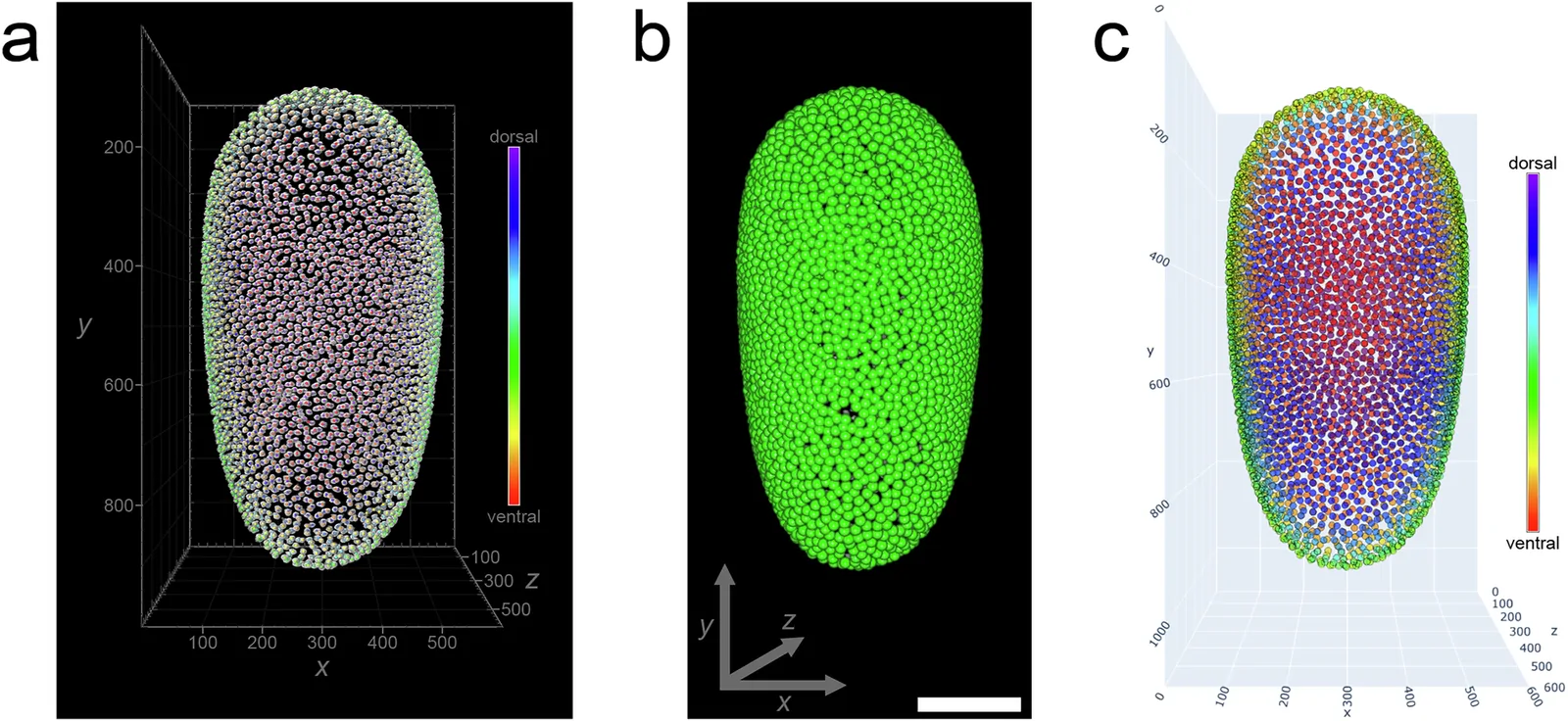
Illustration of nuclei segmentation using three visualization platforms. (**a**) Transparency-based volume rendering in Arivis Pro. This program enables overlay visualization of voxel data (gray-transparent) and the segmented nuclei centroids (ventral-dorsal heatmap). The bounding box indicates voxel dimensions. (**b**) Volume rendering using the 3D Viewer plugin in Fiji. This program also supports overlay visualization of voxel data and centroid positions (green). However, because transparency rendering is not supported, the diameter of the centroid marker spheres must be increased to prevent the markers from disappearing within the nuclei. Heatmap coloring is not supported. Scale bar, 200 pixels. (**c**) Volumetric reconstruction of segmented nuclei centroid positions (ventral-dorsal heatmap) using the Nuclei Pocket Viewer 3D Python script. This program does not support rendering of voxel data. The bounding boxes indicate voxel dimensions. Image data are from dataset DS0005.

For the segmentation data, the SIS files and their accompanying resources can be opened with Arivis Pro version 4.2.1 or higher (Fig. 13a). The XLSX files containing the nuclei coordinates can be used independently or in conjunction with respective image data TIFF files, for example to generate overlays. This can be achieved, for instance, with the 3D Viewer plugin^107^ for Fiji (Fig. 13b). In addition, the Dash web application framework for Python^108^ enables browser-based 3D visualization of nuclei positions with arbitrary rotation (Fig. 13c). A corresponding script is provided (cf. the Code availability section).

Regarding dynamic analyses, the datasets are well suited for, for example, particle image velocimetry-based analyses^109^. The suitability of the datasets for robust long-term single-cell tracking remains to be systematically evaluated.

## Supplementary information

Supplementary Information

Supplementary Information

Supplementary Information

Supplementary Information

## Data Availability

Individual datasets can be accessed via their digital object identifiers^42–104^ (Table 1). Entries belonging to the same dataset are cross-linked to their corresponding parts under ‘Additional details’. An additional entry on Zenodo (https://doi.org/10.5281/zenodo.18015798) allows independent download of the segmentation data^105^.

## Code availability

All Fiji macros used for image processing are available on GitHub (V1.1, https://github.com/BugCube/Kraemer2026A-FijiFusionMacroCollection). The files are numbered according to the processing workflow and additionally tagged to reflect the subfolder structure (cf. the Methods section). Macros labeled ‘Universal’ require no user input, those labeled ‘Parameters’ require user-defined input, and those labeled ‘ParametersDefault’ provide default parameter values that can optionally be modified by the user. Processing parameters are provided in Table 4. The Dash-based Python script for web browser-based 3D visualization of nuclei positions, ‘NucleiPocketViewer3D’, is also available on GitHub (V1.0, https://github.com/BugCube/NucleiPocketViewer3D).

**Table 4:** Image data processing parameters for the Fiji fusion macros.

Parameter	0001	0002	0003	0004	0005	0006	0007	0008	0009	0010	0011	0012	0013	0014	0015	0016
**(1)-[P1-P2]-YZMaximumProjections-V1.1-Parameters.ijm macro**																
Properties: voxel_depth	4	4	4	4	4	4	4	4	4	4	4	4	4	4	4	4
**(2)-[P1-P3]-QCropAndBOut-V1.1-Parameters.ijm macro**																
DR0001 Specify: width	780	740	740	780	780	740	720	720	720	688	720	740	760	740	780	760
DR0001 Specify: x	146	160	160	145	125	150	155	130	160	221	150	140	125	180	115	105
DR0001 Slice Keeper: first	9	47	46	29	36	44	52	35	46	62	49	48	41	35	33	33
DR0001 Slice Keeper: last	203	231	230	223	230	228	231	214	225	233	228	232	230	219	227	222
DR0001 Specify: height	972	940	920	1000	960	935	980	1000	1000	970	1000	950	910	950	950	900
DR0001 Specify: y	104	100	95	65	83	118	118	132	95	78	108	75	140	112	135	127
DR0002 Specify: width	780	740	740	780	780	740	720	720	720	688	720	740	760	740	780	760
DR0002 Specify: x	129	165	145	120	145	160	195	160	165	206	105	125	90	145	120	105
DR0002 Slice Keeper: first	8	40	42	39	34	39	39	52	51	35	35	45	35	34	34	25
DR0002 Slice Keeper: last	202	224	226	233	228	223	218	231	230	206	214	229	224	218	228	214
DR0002 Specify: height	972	940	920	1000	960	935	980	1000	1000	970	1000	950	910	950	950	900
DR0002 Specify: y	116	15	75	80	91	113	130	114	105	99	90	72	120	132	115	140
DR0003 Specify: width	780	740	740	780	780	740	720	720	720	688	720	740	760	740	780	760
DR0003 Specify: x	96	135	165	140	137	145	175	175	155	151	155	125	110	110	125	140
DR0003 Slice Keeper: first	8	48	48	31	32	41	25	35	45	24	38	38	29	34	26	31
DR0003 Slice Keeper: last	202	232	232	225	226	225	204	214	224	195	217	222	218	218	220	220
DR0003 Specify: height	972	940	920	1000	960	935	980	1000	1000	970	1000	950	910	950	950	900
DR0003 Specify: y	126	100	75	85	88	108	112	116	90	99	85	90	110	155	110	137
DR0004 Specify: width	780	740	740	780	780	740	720	720	720	688	720	740	760	740	780	760
DR0004 Specify: x	139	180	150	150	130	135	135	175	160	171	195	140	160	170	120	130
DR0004 Slice Keeper: first	16	31	41	34	30	41	34	19	49	41	49	29	33	46	35	41
DR0004 Slice Keeper: last	210	215	225	228	224	225	213	198	228	212	228	213	222	230	229	230
DR0004 Specify: height	972	940	920	1000	960	935	980	1000	1000	970	1000	950	910	950	950	900
DR0004 Specify: y	112	95	100	70	80	115	102	138	80	80	100	92	130	130	128	124
**(4)-[P4-F1_P5-D1]-AxisAlignmentAndCrop-V1.1-Parameters.ijm macro**																
Rotate: angle (first)	−155	168	166	−178	−138	−165	173	−146	179	−177	−130	147	−155	−168	146	159
Rotate: angle (second)	177	170	177	174	174	−1	−5	−1	178	−1	176	177	173	0	180	178
Rotate: angle (third)	2	−5	2	2	0	−3	2	−6	2	6	−3	1	1	3	−2	4
Specify: width	600	600	600	600	600	600	600	600	600	600	600	600	600	600	600	600
Specify: height	1000	1000	1000	1100	1000	1000	1000	1100	1100	1000	1000	1000	1000	1000	1000	1000
Specify: x	199	143	132	169	139	130	102	126	106	74	196	113	126	98	220	115
Specify: y	354	378	420	342	395	80	96	69	328	52	406	412	369	95	357	376
Slice Keeper: first	181	96	129	143	133	127	91	110	114	52	294	123	120	98	53	128
Slice Keeper: last	780	695	728	742	732	726	690	709	713	651	893	722	719	697	652	727
**(6)-[F1-F2_D1-D2]-YZGradientProjections-V1.1-ParametersDefault.ijm macro (default value was used for all datasets)**																
Custom variable: divisor	3	3	3	3	3	3	3	3	3	3	3	3	3	3	3	3
**(7)-[F3HCorr-F3_D3HCorr-D3]-BrightnessContrastAdjustment-V1.1-Parameters.ijm macro – parameters for the F branch**																
setMinAndMax: min	50	85	85	100	75	75	75	75	60	40	45	40	32	35	35	45
setMinAndMax: max	100	210	190	240	210	175	190	200	100	75	100	75	63	65	80	90
**(7)-[F3HCorr-F3_D3HCorr-D3]-BrightnessContrastAdjustment-V1.1-Parameters.ijm macro – parameters for the D branch**																
setMinAndMax: min	4000	1200	1000	1800	1500	2200	1500	2000	1400	2200	4500	3000	2500	1800	2800	3000
setMinAndMax: max	12000	5000	4000	7500	5500	4000	3500	5500	3000	6000	14000	6000	5500	4000	8000	6500
**(8)-[F3-F4_D3-D4]-YZGradientProjections-V1.1-ParametersDefault.ijm (default value was used for all datasets)**																
Custom variable: divisor	3	3	3	3	3	3	3	3	3	3	3	3	3	3	3	3

These parameters can be used to recreate the intermediate data that were not provided directly, for example the raw fused *z* stacks belonging to the P4 and P5 folders, which were included for only five of the sixteen datasets. Please note that the second macro (‘(2)-[P1-P3]-QCropAndBOut-V1.1-Parameters.ijm’) was executed four times, once for each acquisition direction, using direction-specific parameters.
