# Supplementary material for: Sixteen isotropic 3D fluorescence live-imaging datasets of gastrulation in the red flour beetle Tribolium castaneum (Coleoptera: Tenebrionidae)
Source: Sci Data. 2026 Sep 11;13:1302. doi: 10.1038/s41597-026-08047-9 (PMC13569860; doi:10.1038/s41597-026-08047-9)
Supplement: Supplementary file 1 [file 41597_2026_8047_MOESM4_ESM.pdf]

Supplementary Movie Legends

Supplementary Movie 1 – Representative segmentation of blastodermal nuclei. Transparency-based volume rendering of a fusion-deconvolution-derived 3D image of a *Tribolium* embryo at the uniform blastoderm stage, showing the voxel data in gray-transparent and overlaid with the centroids of the segmented superficial blastodermal nuclei as a ventral-dorsal heatmap. In the first part of the movie, the embryo is rotated around *y*; in the second part, the embryo is rotated around *x*; and in the third part, the embryo is shown from a ventral-anterior oblique perspective. The grid spacing of the bounding box is 100 pixels. Image data are from dataset DS0005. Frame rate is 30 frames per second.

Supplementary Movie 2 – Representative segmentation of serosa nuclei. Transparency-based volume rendering of a fusion-deconvolution-derived 3D image of a *Tribolium* embryo at the onset of germband elongation, showing the voxel data in gray-transparent and overlaid with the centroids of the segmented serosa nuclei as a ventral-dorsal heatmap. In the first part of the movie, the embryo is rotated around *y*; in the second part, the embryo is rotated around *x*; and in the third part, the embryo is shown from a ventral-anterior oblique perspective. The grid spacing of the bounding box is 100 pixels. Image data are from dataset DS0005. Frame rate is 30 frames per second.
