# Supplementary material for: Sixteen isotropic 3D fluorescence live-imaging datasets of gastrulation in the red flour beetle Tribolium castaneum (Coleoptera: Tenebrionidae)
Source: Sci Data. 2026 Sep 11;13:1302. doi: 10.1038/s41597-026-08047-9 (PMC13569860; doi:10.1038/s41597-026-08047-9)
Supplement: Supplementary file 2 [file 41597_2026_8047_MOESM1_ESM.docx]

**Supplementary Figure 1 – Dataset-resolved overview of developmental staging.** Green highlighting indicates developmental time points at which blastodermal nuclei (first column) or serosa nuclei (fifth column) were segmented. The uniform blastoderm stage is absent from DS0001 and DS0003 since imaging commenced after this stage had already passed. Scale bar, 100 µm.
